# Supplementary material for: Automated classification of estrous stage in rodents using deep learning
Source: Sci Rep. 2022 Oct 21;12:17685. doi: 10.1038/s41598-022-22392-w (PMC9587051; doi:10.1038/s41598-022-22392-w)
Supplement: Supplementary file 1 — Supplementary Information. [file 41598_2022_22392_MOESM1_ESM.docx]

**Supplementary Material for:**

**Automated classification of estrous stage in rodents using deep learning**

Nora S. Wolcott^1^, Kevin K. Sit^2^, Gianna Raimondi^3^, Travis Hodges^4^, Rebecca M. Shansky^5^, Liisa A. M. Galea^4,6^, Linnaea E. Ostroff^3^, Michael J. Goard^1,2,7, *^

*Correspondence to: [michael.goard@lifesci.ucsb.edu](mailto:michael.goard@lifesci.ucsb.edu)

**Supplementary Figures S1-S4**

**Supplementary Tables S1-S2**


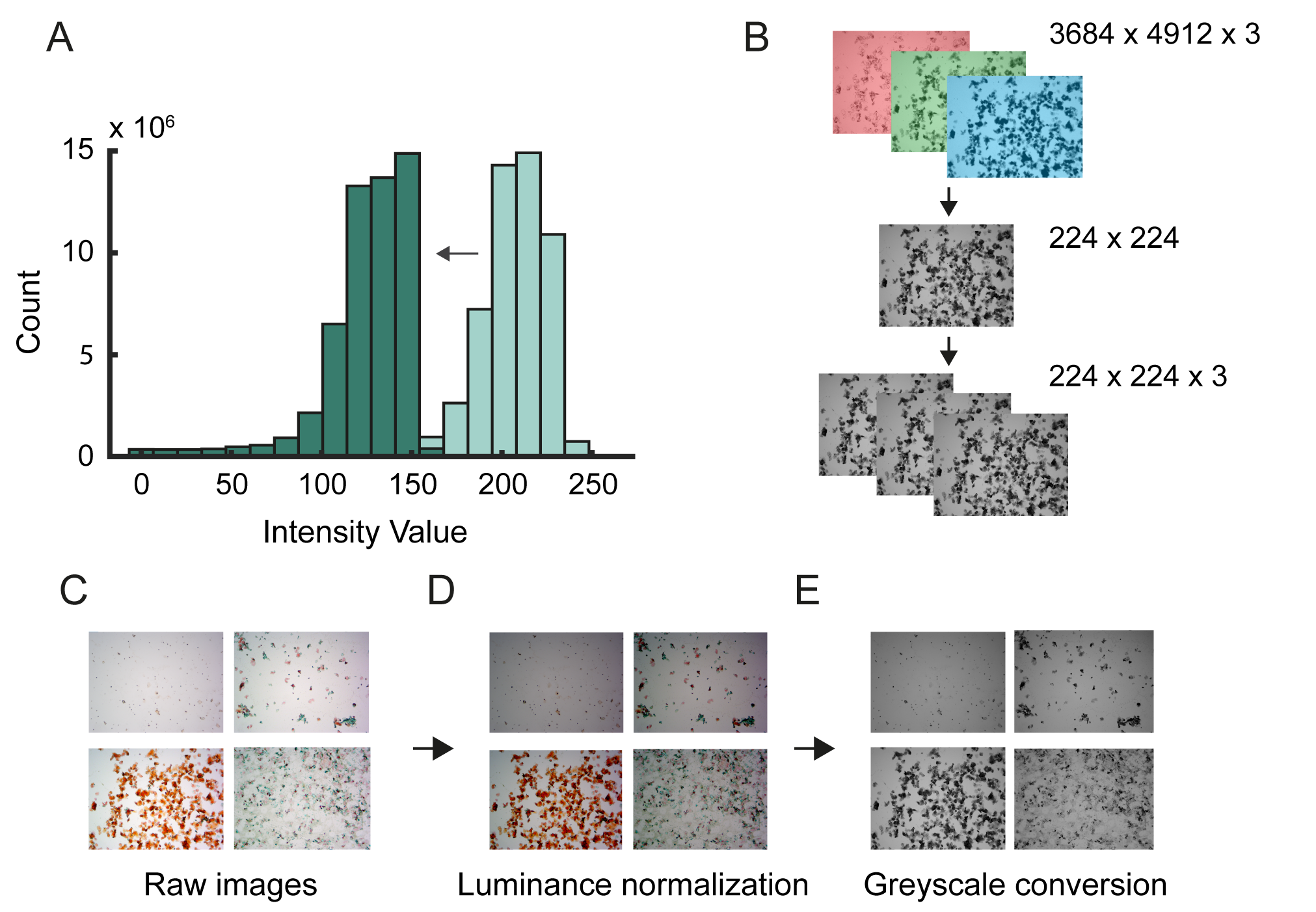


**Supplementary Figure S1.** **Image preprocessing pipeline.**

1. Intensity histogram of an unprocessed image (light blue), shifted to lower intensity (dark blue) during luminance normalization.
2. Schematic of image resizing and conversion to grayscale, where 1D grayscale images are concatenated into a 3D array of size 224 x 224 x 3 to match the input requirements of the transfer learning network.
3. Example unprocessed test images from one estrous cycle.
4. Raw images with reduced intensity, normalized to the same maximum intensity peak.
5. Luminance-normalized images converted to 3-channel grayscale.


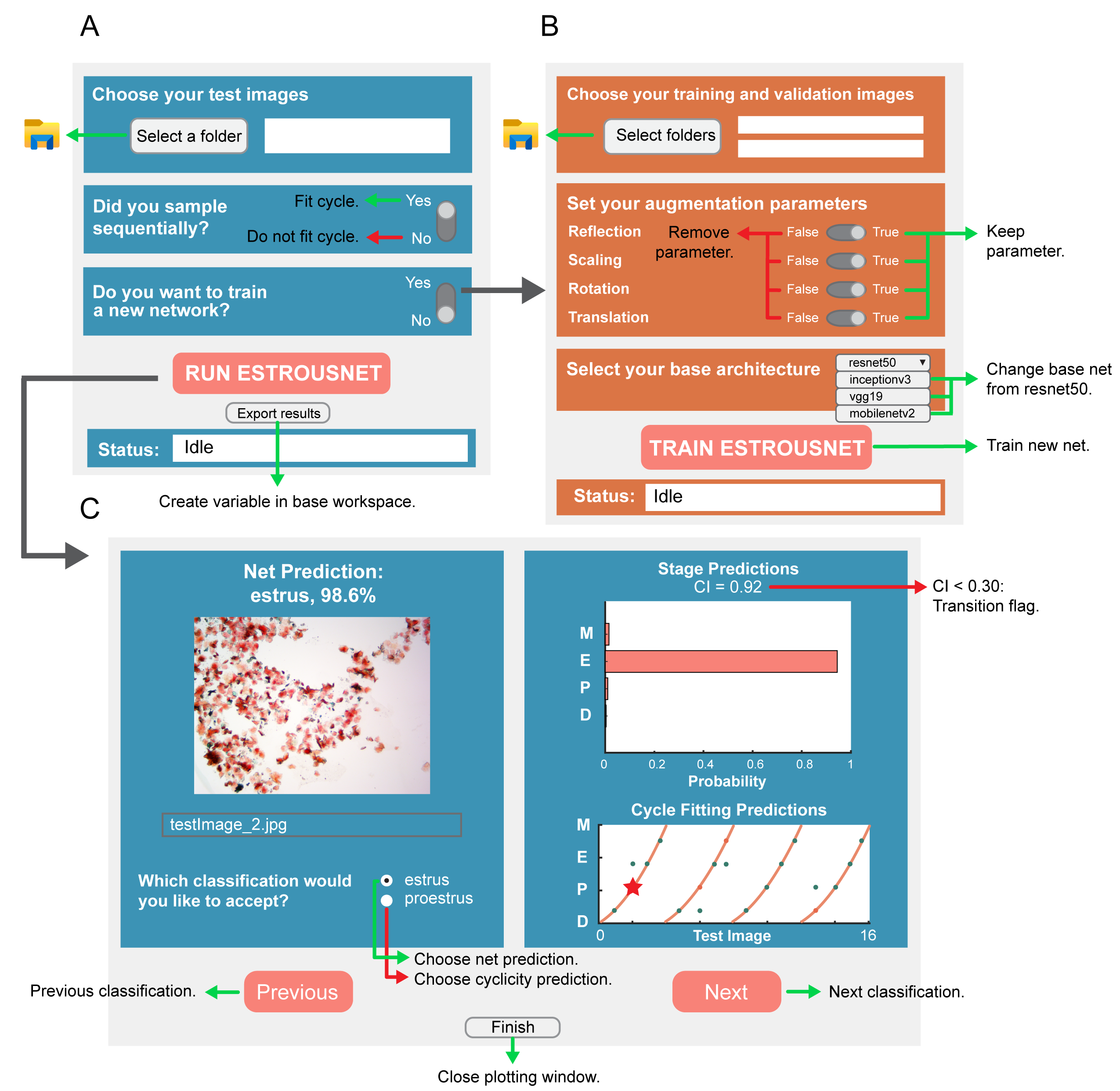


**Supplementary Figure S2.** **Illustration of the EstrousNet user interface (GUI).**

1. The EstrousNet classification GUI: the user selects a folder of test images which are automatically classified and plotted. The user also selects whether images were sampled sequentially, which will determine whether net classifications are fit to an archetypical cycle.
2. The EstrousNet training GUI: if the user selects that they would like to train a new network, it will launch the training GUI. This GUI lets the user select folders with training and validation images, as well as custom augmentation parameters, and once training is finished will save the trained network and training data to the current directory.
3. The EstrousNet plotting GUI: once the classification GUI is used to select test images, the plotting GUI will display the results of the net classifications, along with the probabilities of each individual stage and the confidence index (CI) based off these probabilities. If images were taken in sequence, the plotting GUI will fit the images to an archetypal cycle, and for any images where the cyclicity and net classifications disagree, the user can choose to manually select the preferred classification.


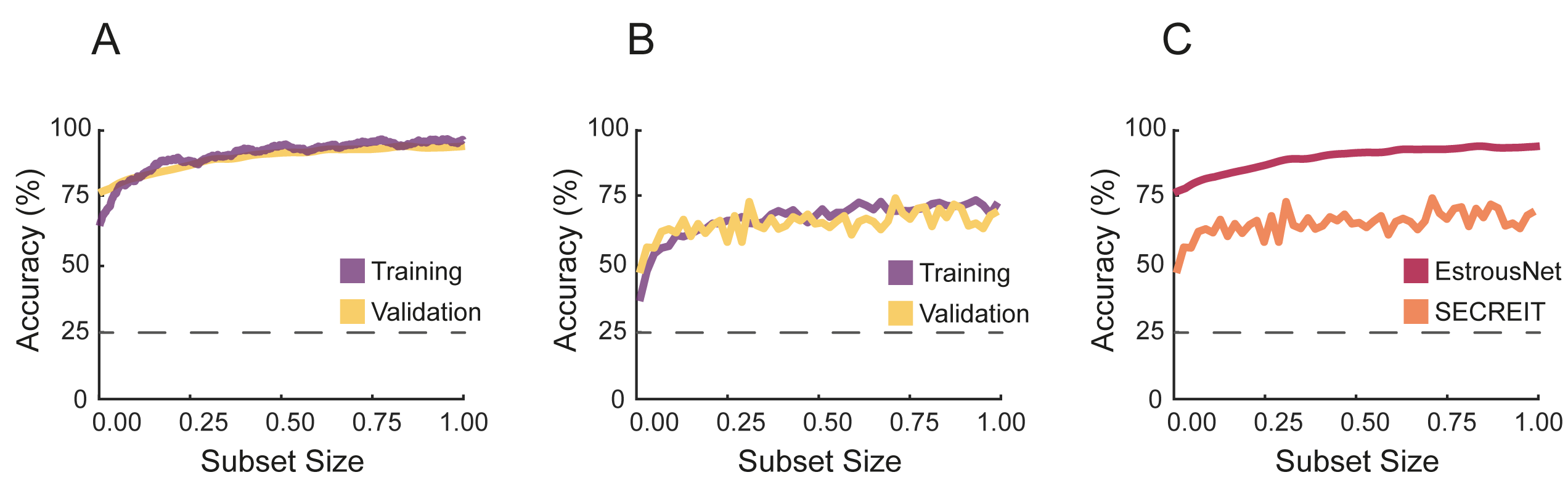


**Supplementary Figure S3.** **Training and validation curves for EstrousNet compared to previous models.**

1. Training and validation curves for EstrousNet trained on EstrousBank. Dotted line indicates chance floor (25%).
2. Training and validation curves for SECREIT^12^ trained on EstrousBank. Dotted line indicates chance floor (25%).
3. Validation curves for EstrousNet (pink) and SECREIT (orange) trained on EstrousBank, plotted against each other. Grey dotted line indicates chance floor (25%).


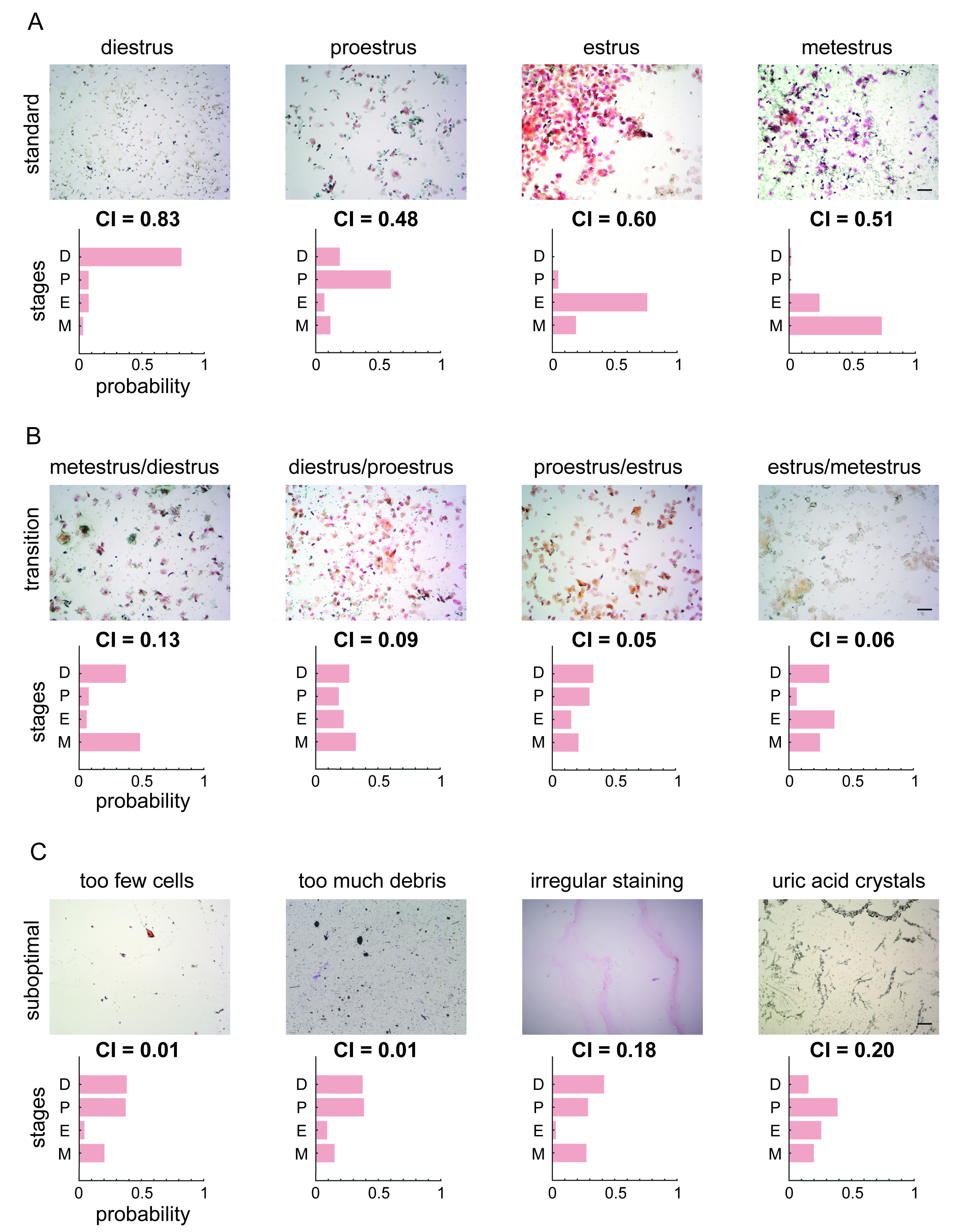


**Supplementary Figure S4.** **Confidence indices for standard, transition, and suboptimal images.**

1. Four Shorr stained murine cytology images taken 24hrs apart spanning the standard archetypal estrous stages: diestrus, proestrus, estrus, and metestrus. Bar charts show the probability, given by EstrousNet, of the images belonging to one of the four estrous stages (D = diestrus, P = proestrus, E = estrus, M = metestrus). CI, or confidence index, indicates the degree of certainty of the image belonging to the given stage. Scale bar = 10 μm.
2. Four Shorr stained murine cytology images taken 24 hrs apart spanning four transition stages: metestrus/diestrus, diestrus/proestrus, proestrus/estrus, and estrus/metestrus. Bar charts show the probability, given by EstrousNet, of the images belonging to one of the four standard estrous stages (D = diestrus, P = proestrus, E = estrus, M = metestrus). CI, or confidence index, indicates the degree of certainty of the image belonging to the given stage. Scale bar = 10 μm.
3. Four cytology images deemed suboptimal for estrous stage classification due to different criteria: too few cells, too much debris, irregular Shorr staining, and a prevalence of uric acid crystals. Bar charts show the probability, given by EstrousNet, of the images belonging to one of the four standard estrous stages (D = diestrus, P = proestrus, E = estrus, M = metestrus). CI, or confidence index, indicates the degree of certainty of the image belonging to the given stage. Scale bar = 10 μm.

| **Source Lab** | **Magnification** | **Stain** | **Species** | **Strain** | **# images** | **% of total images** |
| --- | --- | --- | --- | --- | --- | --- |
| Galea | 20X | Cresyl Violet | Rat | Sprague Dawley WT | 145 | 1.14 |
| Goard | 10X | H&E | Mouse | Thy1-GFP-M (Jax Stock #007788), Slc7a7-cre (Jax Stock #023527) x TITL-GCaMP6s (Jax Stock #024104) C57BL/6J | 260 | 2.04 |
| Goard | 10X | Shorr Stain | Mouse | Thy1-GFP-M (Jax Stock #007788), Slc7a7-cre (Jax Stock #023527) x TITL-GCAMP6s (Jax Stock #024104) C57BL/6J | 764 | 6.01 |
| Ostroff | 10X | H&E | Rat | Sprague Dawley WT | 2091 | 16.44 |
| Ostroff | 10X | Shorr Stain | Rat | Sprague Dawley WT | 4186 | 32.91 |
| Shansky | 10X | Crystal Violet | Rat | Long Evans WT | 1954 | 15.36 |
| Sutoh | 10X | Giemsa | Mouse | C57BL/6J WT | 3319 | 26.09 |

Supplementary Table S1. **Summary of EstrousBank images from multiple labs.**

Metrics for the images included in the open-source image repository EstrousBank, subdivided by the groups contributing the raw images.

|  | **X (% of total cycle)** | **X (days)** | **Y** |
| --- | --- | --- | --- |
| **Diestrus** | 21.63 | 1.04 | 0.50 |
| **Proestrus** | 51.22 | 2.47 | 1.50 |
| **Estrus** | 69.87 | 3.39 | 2.50 |
| **Metestrus** | 90.27 | 4.39 | 3.50 |

Supplementary Table S2. **Coordinates of archetypal estrous cycle waveform.**

X and Y coordinates for the estrous cycle waveform used for cycle fitting, based on reporting of cycle length from 12 groups ^10,18,20–23,30,35–38^. X coordinates are the midpoint of each estrous cycle stage, reported as a % of the total average cycle length (4.8 days), as well as in days.

­­
